# Cloud-Based Quad Deep Ensemble Framework for the Detection of COVID-19 Omicron and Delta Variants

**DOI:** 10.3390/diagnostics13223419

**Published:** 2023-11-09

**Authors:** Ravi Shekhar Tiwari, Lakshmi Dandabani, Tapan Kumar Das, Surbhi Bhatia Khan, Shakila Basheer, Mohammed S. Alqahtani

**Affiliations:** 1 Department of Computer Science Engineering, Mahindra University, Hyderabad 500043, India; 2 School of Computing Science and Engineering, VIT Bhopal University, Bhopal 466114, India; lakshmi.d@vitbhopal.ac.in; 3 School of Computer Science Engineering and Information Systems, Vellore Institute of Technology, Vellore 632014, India; 4 Department of Data Science, School of Science Engineering and Environment, University of Salford, Manchester M5 4WT, UK; 5 Department of Engineering and Environment, University of Religions and Denominations, Qom 13357, Iran; 6 Department of Electrical and Computer Engineering, Lebanese American University, Byblos P.O. Box 13-5053, Lebanon; 7 Department of Information Systems, College of Computer and Information Science, Princess Nourah Bint Abdulrahman University, P.O. Box 84428, Riyadh 11671, Saudi Arabia; sbbasheer@pnu.edu.sa; 8 Radiological Sciences Department, College of Applied Medical Sciences, King Khalid University, Abha 61421, Saudi Arabia; mosalqhtani@kku.eu.sa; 9 BioImaging Unit, Space Research Centre, Michael Atiyah Building, University of Leicester, Leicester LE1 7RH, UK

**Keywords:** COVID-19, Omicron variant, Delta variant, transfer learning, stacking, deep learning, weighted-average ensemble

## Abstract

The mortality rates of patients contracting the Omicron and Delta variants of COVID-19 are very high, and COVID-19 is the worst variant of COVID. Hence, our objective is to detect COVID-19 Omicron and Delta variants from lung CT-scan images. We designed a unique ensemble model that combines the CNN architecture of a deep neural network—Capsule Network (CapsNet)—and pre-trained architectures, i.e., VGG-16, DenseNet-121, and Inception-v3, to produce a reliable and robust model for diagnosing Omicron and Delta variant data. Despite the solo model’s remarkable accuracy, it can often be difficult to accept its results. The ensemble model, on the other hand, operates according to the scientific tenet of combining the majority votes of various models. The adoption of the transfer learning model in our work is to benefit from previously learned parameters and lower data-hunger architecture. Likewise, CapsNet performs consistently regardless of positional changes, size changes, and changes in the orientation of the input image. The proposed ensemble model produced an accuracy of 99.93%, an AUC of 0.999 and a precision of 99.9%. Finally, the framework is deployed in a local cloud web application so that the diagnosis of these particular variants can be accomplished remotely.

## 1. Introduction

Numerous people’s health all across the world was impacted by COVID-19. We have not yet been shielded from the coronavirus health dangers, even after three years of pandemic. This is mostly because of the variant mutations, which increasingly pose challenges in terms of treatment. The SARS-CoV-2 Delta variant (B.1.617.2) that evolved from the outbreak in India has fueled the spread of the outbreak in several developed countries [1]. In the initial days of the pandemic in 2020, South East Asia and Central Asia were the role models in terms of COVID-19 control and containment. However, the scenario had changed by mid-2021, with a million cases reported daily from Asia only. India’s COVID-19 deaths added up to 259,302 in 2021 compared to 148,738 in the year 2020. This is mostly due to the incident of the Delta variant in 2021. The second wave of COVID-19, driven by Omicron and Delta variants, brought about a severe crisis and caused the collapse of healthcare systems in Central Asia and the Middle East [2]. This was the time when the COVID-19 pandemic was beginning to recede in other parts of the world. In order to cope with the exponential rise in infections, researchers and experts began working on enhancing symptomatic testing due to the need for quicker and less expensive diagnostic approaches. However, prediction and treatment remain difficult, owing to their unusual varieties. Three clinical tests, notably the RT-PCR test, the X-ray, and the CT scan, are useful for diagnosing, treating, and isolating patients in order to prevent further transmission. Numerous studies have been conducted using radiological imaging to determine COVID-19’s detection ability. According to the research, chest CT scanning has a lower rate of false-positives than X-ray imaging classification [3]. Hence, automatic COVID-19 detection from chest CT scans is essential for a fast and precise diagnosis.

Machine learning (ML) and deep learning models are quite effective in classifying chest scans in order to detect a disease. However, deep learning models are preferred for their inherent abilities to extract features automatically and to deal with a huge number of features present in the images. In deep learning, both transfer learning and ensemble models have ushered in significant advancements, most notably in the field of computer vision. When confronted with an identical computer vision problem, these pre-trained models can be leveraged rather than going through the lengthy process of training models from scratch [4]. Transfer learning is an important technique that has demonstrated considerable benefits in computer vision and a variety of other fields as well. Transfer learning models are advantageous since they permit training on a smaller dataset and also help to reduce training time with a few changes to the architecture [5]. With an optimum initial starting point, transfer learning models deliver outputs with a higher degree of accuracy. Transfer learning alleviates the need for time-consuming and intensive data collection, cleaning, labeling, and training methods [6].

Deep learning models based on ensemble techniques are widely used within the field of image classification [7]. Various methods exist for combining or aggregating classifiers into an ensemble. On the other hand, there are two primary options for transferring knowledge using a pre-trained model. One is model fine-tuning, where extra short-term training is provided to the original model in order to include a specific training set in the model’s knowledge base. However, the second option is to utilize a pre-trained CNN as a feature extractor to convert photos to feature vectors suitable for classification [8,9,10].

The following are the highlights of our contributions:

- An ensemble-based deep transfer learning framework is designed for diagnosing Delta and Omicron variants from CT-scan images.
- We used three deep transfer learning models, i.e., VGG-16, Densenet-121, and Inception-v3, and one CapsNet model during the process of stacking and weighed averaging.
- Our proposed approach generated an overwhelming accuracy of 99.93% over the validation and test data.
- The developed framework is deployed in a cloud-based web application.

The rest of this paper is organized as follows: in Section 2, we review the articles pertaining to COVID-19 identification from CT scan and chest X-rays; the detailed methodology and techniques adopted are briefed in Section 3; the performance of transfer learning and other base learners is discussed in Section 4; and Section 5 exhibits the experimental results obtained using the ensemble methods along with their analysis. The paper is concluded in Section 6.

## 2. Related Work

Coronavirus infection is primarily a respiratory disease. Hence, the focus of most studies is to determine the presence of COVID infection in the lungs. This section consolidates recent studies on the classification of CT-scan images of the lungs infected by COVID-19 Omicron and Delta variants utilizing ensemble approaches and transfer learning techniques. After examining the relevant literature from 2020 to 2022 including variants like SARS-CoV-2 and COVID-19 (Delta type, Omicron, etc.), and analyzing their strengths and weaknesses, we summarize the key points of each article in Table 1.

### 2.1. Artificial Intelligence in Healthcare

In this section, we present the state-of-the-art AI applications in disease diagnosis and detection, as well as challenges in the adoption of AI in healthcare.

Meskó et al. [22] recommend the integration of large language models (LLMs) into medical practice. They advocate for regulatory guidance for companies and healthcare organizations on the seamless deployment of LLMs into existing products and services. The framework should encompass not only text-based interactions but also potential future applications like sound or video analysis. Morais et al. [23] introduce a novel approach to breast cancer diagnosis using magnetic resonance imaging (MRI). The framework employs a 3D-CNN for each modality, and predictions are integrated using a late fusion strategy based on the Dempster–Shafer theory. The study demonstrates that the combined analysis of multiple modalities enhances overall diagnostic accuracy compared to individual modalities.

Alkan et al. [24] provide various means and measures to enhance the interactivity of AI solutions and strategies for resilient models in healthcare, such as human-centered computing, human–computer interaction (HCI), and interactive systems and tools. Calisto et al. [25] detail the field research, design, and comparative implementation of a multimodal user interface for medical imaging in breast screening. The article concludes by summarizing the findings and offering recommendations from radiologists to guide the future design of medical imaging interfaces.

Lin et al. [26] present the contemporary utilization of AI in radiosurgery, specifically exploring its application in stereotactic radiosurgery. The review encompasses various facets of stereotactic radiosurgery where ML and deep learning models have been employed. Diogo et al. [27] address the complexities introduced by 3D MRI for breast cancer lesions, such as data scarcity and a lack of local annotations, using a novel two-stage framework.

Donins et al. [28] explore the challenges and potential solutions associated with the adoption of AI in healthcare. The study identifies key challenges such as the lack of transparency in AI decision-making, concerns about data privacy and security, and hesitancy among healthcare professionals and patients. Calisto et al. [29] investigate how factors such as security, risk, and trust influence the acceptance of AI-based assistance in medical contexts.

Sivaraman et al. [30] present a framework for a decision support interface that offers interpretable treatment suggestions for sepsis, a critical condition characterized by decisional uncertainty, varying treatment practices, and potentially poor outcomes, even with optimal decisions. Calisto et al. [31] introduce BreastScreening-AI, a novel approach for classifying multimodal breast images within two scenarios: Clinician-Only and Clinician-AI. The Clinician-AI scenario exhibits superiority and also reduces the time to diagnose by 3 min per patient.

Bone shadow suppression without distorting the spatial features of chest X-ray images is studied by Rani et al. [32]. They developed the spatial feature and resolution maximization (SFRM) GAN to efficiently minimize the visibility of bones in chest X-rays while ensuring the maximum retention of critical information.

### 2.2. Findings from Literature Review

The literature claims that DenseNet-201 is an extremely effective deep neural network design for medical imaging, especially for feature extraction [33,34].

There are several transfer learning models that are taken into consideration, including ImageNet, VGG-16, DenseNet, and Xception. However, only the VGG-16 [35], DenseNet [36], and Xception [37] models are preferred because of their popularity and higher accuracy.

The characteristics of a feature, such as its orientation, size, velocity, or color, are not taken into account by neurons. The deep neural network’s CapsNet algorithm preserves precise posture data. In a small, unbalanced dataset, CapsNet performed better than the other networks. CapsNet excels at handling the challenge of image classification and outperforms its peers in terms of accuracy and parameter counts [38].

The main flaw with the convolutional neural network (CNN) model is that its neurons are activated depending on whether they are likely to detect specific features. The characteristics of a feature, such as direction, size, velocity, and color, are ignored by neurons. All neural networks in CapsNet keep accurate posture information. As a result, this particular model is preferred.

## 3. Methodology

In this section, the detailed experimental methodology of the dataset, the CNN models used, and the results obtained are discussed.

### 3.1. Dataset

In this research of detecting COVID-19 Omicron and Delta variants, a dataset of lung CT-scan images is used. We collected the dataset from the Kaggle platform for the binary classification of the CT-scan lung images (COVID-positive or COVID-negative) [39]. The dataset was collected from realistic patient records at radiology clinics affiliated with teaching hospitals in Tehran, Iran. Some of the CT scans have a date and sequence number engraved. We implemented erosion and dilation pre-processing techniques to avoid biases while training the model. However, the CT scans were collected from real patients in radiology centers of teaching hospitals in Tehran. Hence, there could be demographic biases such as geographic location, socioeconomic factors, health behaviors and beliefs, and patient referral patterns.

Table 2 shows the distribution of the dataset for the two distinct labels: COVID-positive and COVID-negative.

**Figure 1:**
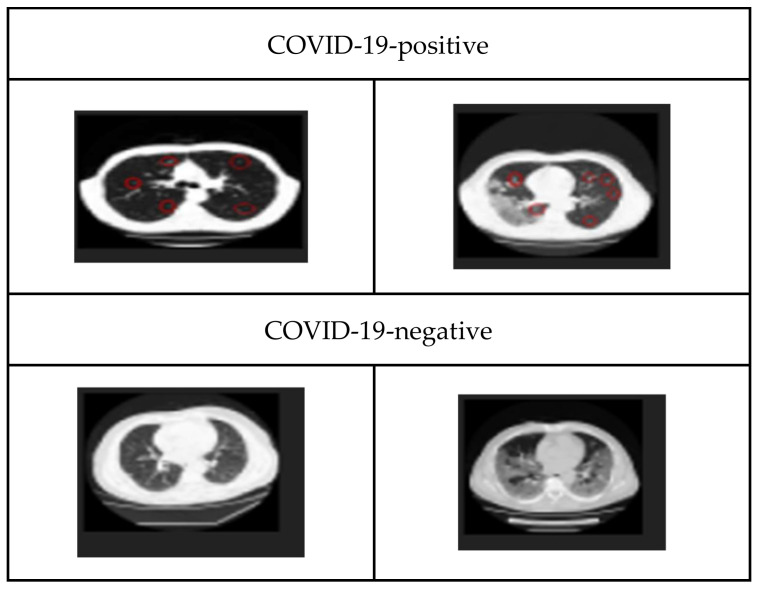
CT-scan images—COVID-19-positive and COVID-19-negative from the Kaggle CT-scan dataset.

A few snapshots of COVID and non-COVID images are exhibited in Figure 1.

### 3.2. The Proposed Architecture

These CT scans have some data engraved on them, such as date and sequence number, which could lead to data leakage while training the model. In order to avoid the data leakage scenario, we executed the erosion pre-processing technique on the bottom right side of the image so that this information could be removed from the CT-scan images, and after that, we implemented a dilation pre-processing technique. Finally, we rescaled the image between 0 and 1 by pixel rescaling. Three pre-processing approaches, including erosion, dilation, and pixel rescaling, were implemented in order to remove such undesired information from the images [40].

**Figure 2:**
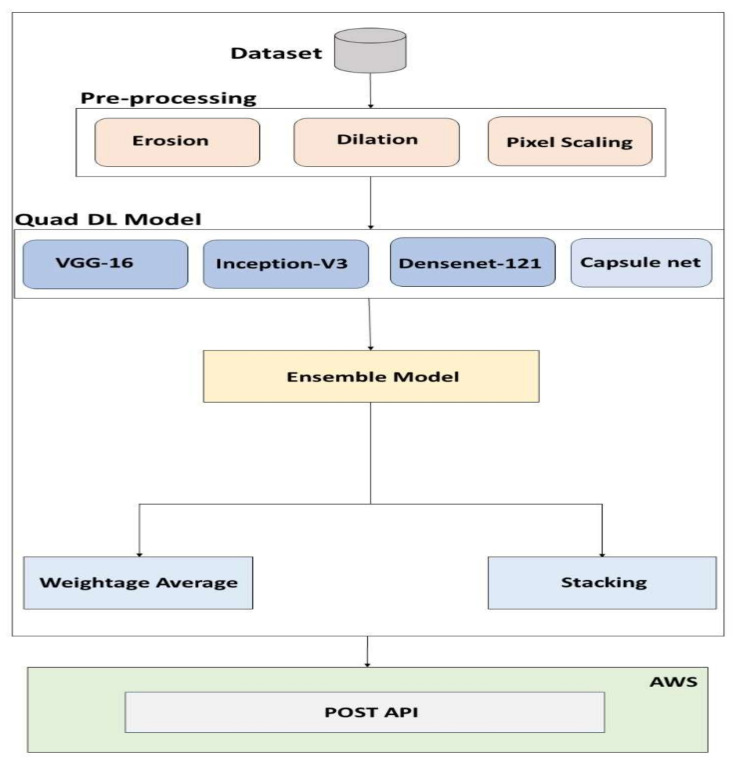
Proposed ensemble framework and model deployment.

The proposed design of the deep learning architecture consists of CapsuleNet; ensemble models such as VGG-16 and Xception act as the basis learners in the proposed ensemble architecture; and Classifier ANN performs as an aggregator. The CNN’s weakness is that its neurons are engaged based on their likelihood of recognizing specific features. In the proposed ensemble architecture, CapsuleNet and ensemble models such as VGG-16 and Xception serve as the basic learners, whereas Classifier ANN acts as an aggregator. Several transfer learning models, including ImageNet, VGG-16, DenseNet, and Xception, are considered; however, only the VGG-16, DenseNet, and Xception models are considered because of their superior accuracy. Each of these models was trained by utilizing k-fold training techniques on the dataset. Figure 2 shows the proposed methodology diagrammatically.

**Figure 3:**
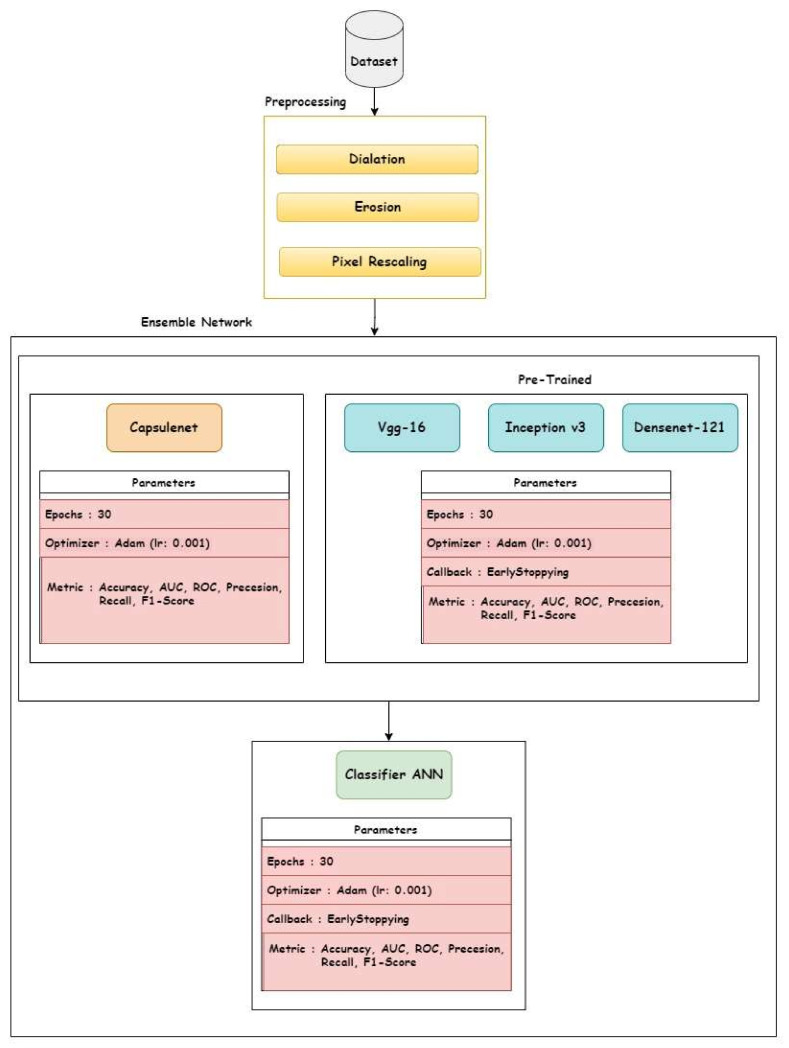
Proposed methodology for the transfer learning-based ensemble architecture.

Figure 3 represents the proposed framework of the ensemble-based approach. We made use of transfer learning models, namely VGG-16, Densenet-121, and Inceptionv3, as base learners, and CapsuleNet. The rationale behind adopting transfer learning models as base learners in a 3:1 ratio is to overcome the issue of insufficient datasets.

Each of the pre-trained models has exactly the same hyperparameters. The details are given in the results section. However, in CapsuleNet, the number of filters (512, 256, 128, 64) used is different but the other hyperparameters remain the same. For the aggregator model, a simple ANN is used as the base model but with a different number of neurons, and the aggregator ANN was trained on the accumulated output from the base model with the original labels of the CT-scan dataset.

### 3.3. Model Deployment

A web-based application was developed to deploy the framework in a cloud platform. Deploying a Django Rest API that accepts image uploads on AWS involves several crucial steps to ensure smooth functionality. First, an Amazon EC2 instance with the required resources was set up, which granted the necessary permissions for the Django application to run effectively. Next, the Django system was deployed onto the instance, transferring our codebase via Git, SCP, or Amazon S3. Then, the web server was configured to serve the Django application, configuring reverse proxy settings to direct incoming HTTP requests. For input, Amazon S3 was employed for storage and retrieval, adapting Django settings to use the S3 storage backend.

### 3.4. Validation Metrics

To confirm the performance of the proposed ensemble-based transfer learning model, the confusion matrix and the associated parameters, e.g., Accuracy, F1 score, Precision, and Recall, were taken into consideration as performance metrics [41].

(1) $$Accuracy=\frac{\left(TP+TN\right)}{\left(TP+FN+FP+TN\right)}$$

(2) $$Recall=\frac{\left(TP\right)}{TP+FN}$$

(3) $$\mathrm{Precision}\;=\;\frac{\left(TP\right)}{TP+FP}$$

(4) $$F1-Score=\frac{2\cdot \left(Recall\cdot \mathrm{Precision}\right)}{Recall\cdot \mathrm{Precision}}$$

where TP = True-Positive, TN = True-Negative, FP = False-Positive and FN = False-Negative.

## 4. Performances of Transfer Learning Models

The total dataset consists of 14,182 images; the dataset was split into three parts based upon the count of positive and negative samples, such that the training set consists of 11,586 images; the validation set consists of 1448 images, and the test set consists of 1448 images. All the models were trained on the AWS EC2, and the dataset was stored in AWS S3 so that it would be efficient and easy to implement. AWS EC2 is powered by Intel 8th Generation, 4.5 GHz Turbo, i7 Core, and has six physical processors, twelve logical processors, and 32 GB memory. We developed the code of transfer learning and ensemble models using Python 3.8 Keras API, whereas the CapsNet model was developed using Python 3.8 Pytorch libraries.

### 4.1. VGG-16 Model

**Figure 4:**
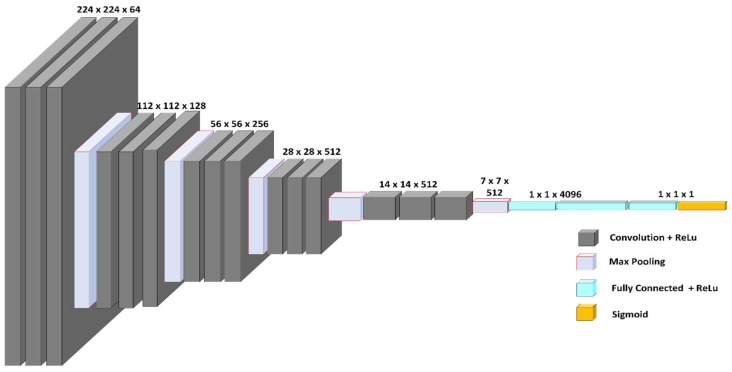
VGG-16 model architecture.

**Figure 5:**
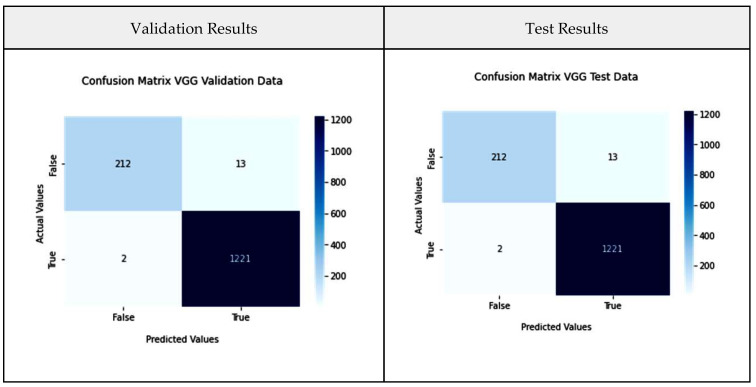

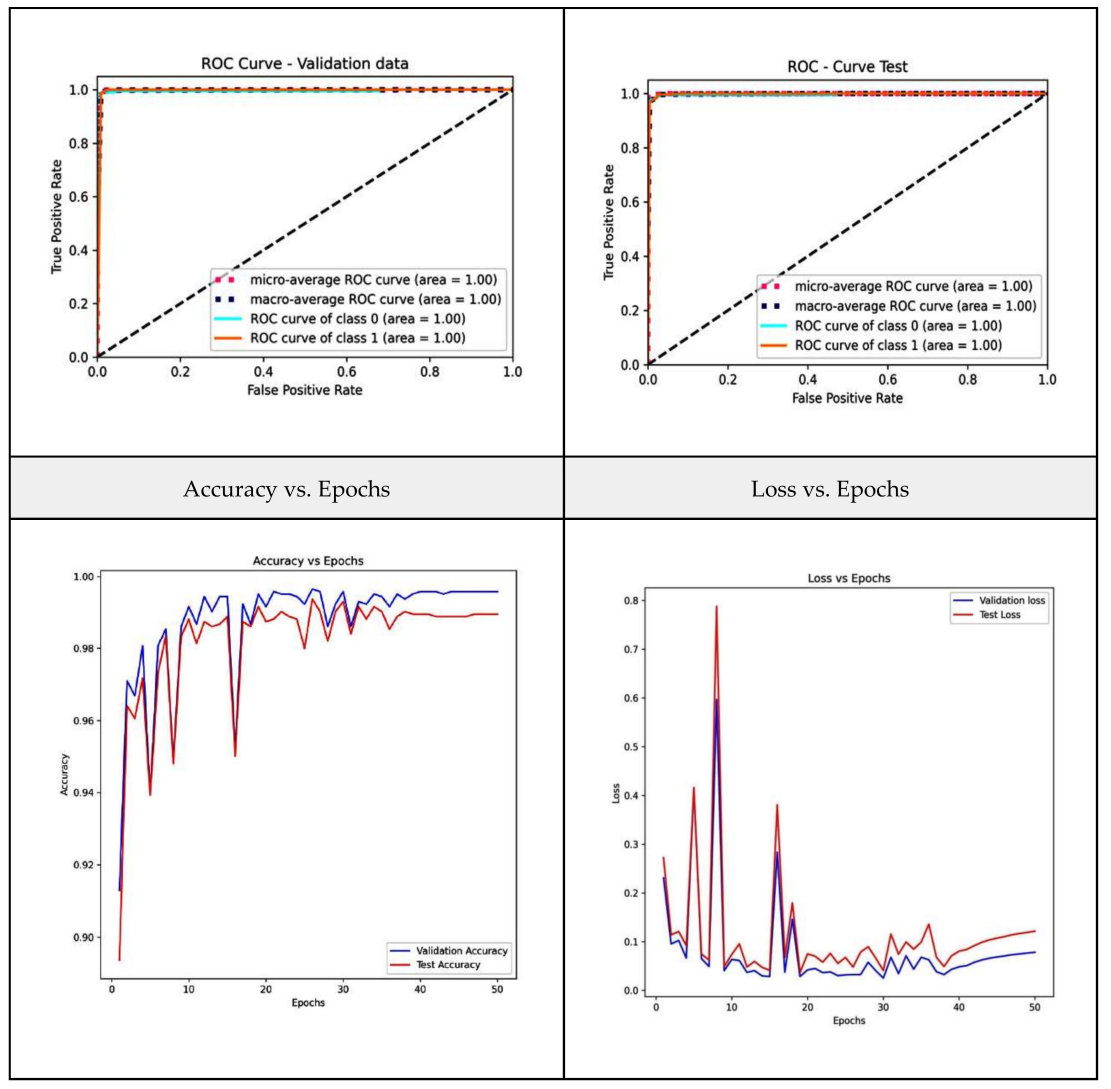
VGG-16 model performance metrics.

VGG-16 consists of five blocks and three levels that are all interconnected. Each block consists of several convolutional layers and a max-pooling layer. After flattening the output matrix after block 5, there are two layers for binary classification that are fully connected [42], and this is represented in Figure 4. In our current study, VGG-16 represents the base model; it is fed with the CT-scan images and original labels. In a later phase, its output is used as an input to the aggregator model. Figure 5 shows the VGG-16 model performance metrics such as the confusion matrices and the ROC curve for the validation and test data separately, along with the graphs for the variation in accuracy and loss over the number of epochs.

The pre-trained model VGG-16 weights are not frozen, i.e., they are not optimized during the training phase. The model was trained using Adam (lr = 0.001), dropout (probability = 0.2), and a dense layer of (1000, 75, 2). We used callbacks that have a patience of 5 and monitored value_loss while training. After training over these parameters, the best model metrics are listed below:

- Epochs: 8/30.
- Callbacks: Early stopping on val_accuracy with patience 5, restore best weights true.
- Optimizer: Adam with a learning rate of 0.001.
- Loss: Binary Cross Entropy without logits.
- Image_size: (224 × 224 × 3), type: tensor.
- Dense Layer Weight—Initializer: glorot_uniform.
- Activation: Relu.
- Use Bias: True.
- Dense Layer Bias—Initializer: zeros.
- Batch Normalization: a momentum of 0.99 with an epsilon of 0.001 along with a gamma-initializer of ones, a beta-initializer of zeros, and a moving mean initializer of zeros.

From the confusion matrix, it is evident that only 15 samples are misclassified out of 1448 scans. The model achieved an average accuracy of 98.2 percent and an AUC value of 0.992. Initially, accuracy increased sharply until 10 epochs, further between 10 and 30 epochs, varied a bit after 40 epochs, and the accuracy almost became stable around the value of 0.985d.

### 4.2. Densenet-121 Model

**Figure 6:**
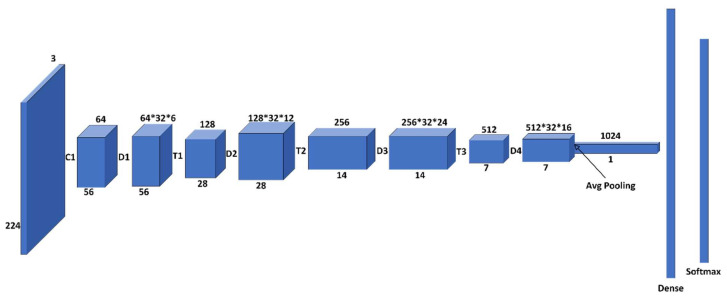
Densenet-121 model architecture.

Densenet-121 consists of five blocks and three levels, and all are interconnected. Each block consists of several convolutional layers and a max-pooling layer. After flattening the output matrix after block 5, there are two layers for binary classification that are fully connected [43], and this is shown in Figure 6.

**Figure 7:**
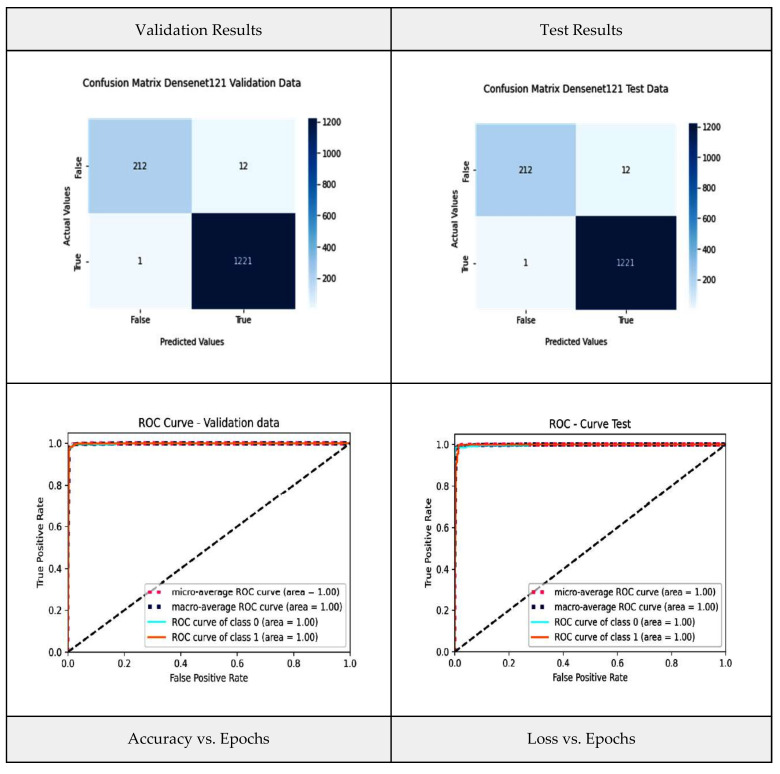

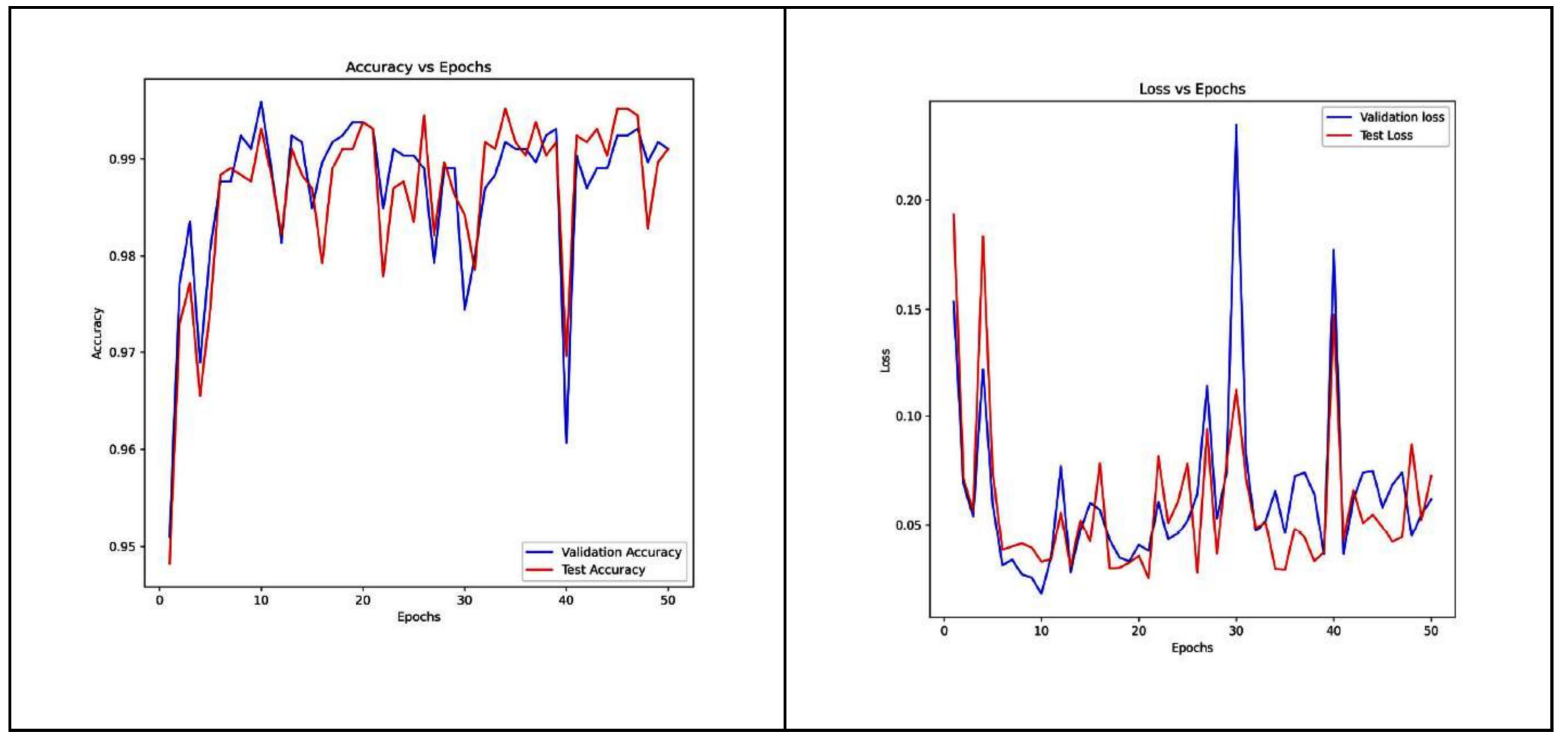
Densenet-121 model performance metrics.

In our research study, Densenet-121 represents the base model that accepts the CT-scan images and original labels. In a later phase, its output is used as an input in the aggregator model. Figure 7 shows the Densenet model performance metrics, such as the confusion matrix and the ROC for the validation and test data, as well as graphs for accuracy vs. epochs and loss vs. epochs.

The model was trained using Adam (lr = 0.001), dropout (probability = 0.2), and a dense layer of (1000, 75, 2). We used callbacks that have a patience of 5 and monitored value_loss while training. After training over these parameters, the best model metrics are:

- Epochs: 8/30.
- Callbacks: Early stopping on val_accuracy with patience 5, restore best weights true.
- Optimizer: Adam with learning rate of 0.001.
- Loss: Binary Cross Entropy without logits.
- Image_size: (224, 224, 3) type: tensor.
- Dense Layer Weight—Initializer: glorot_uniform.
- Activation: Relu.
- Use Bias: True.
- Dense Layer Bias—Initializer: zeros.
- Batch Normalization: a momentum of 0.99 with an epsilon of 0.001 along with a gamma-initializer of ones, a beta-initializer of zeros and a moving mean initializer of zeros.

### 4.3. Inception-V3 Model

**Figure 8:**
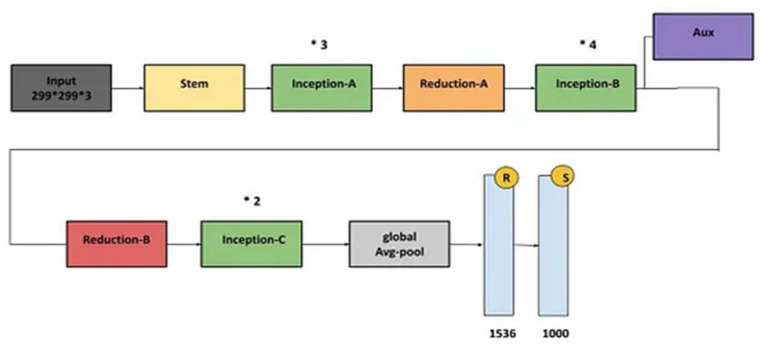
Inception-v3 model architecture.

**Figure 9:**
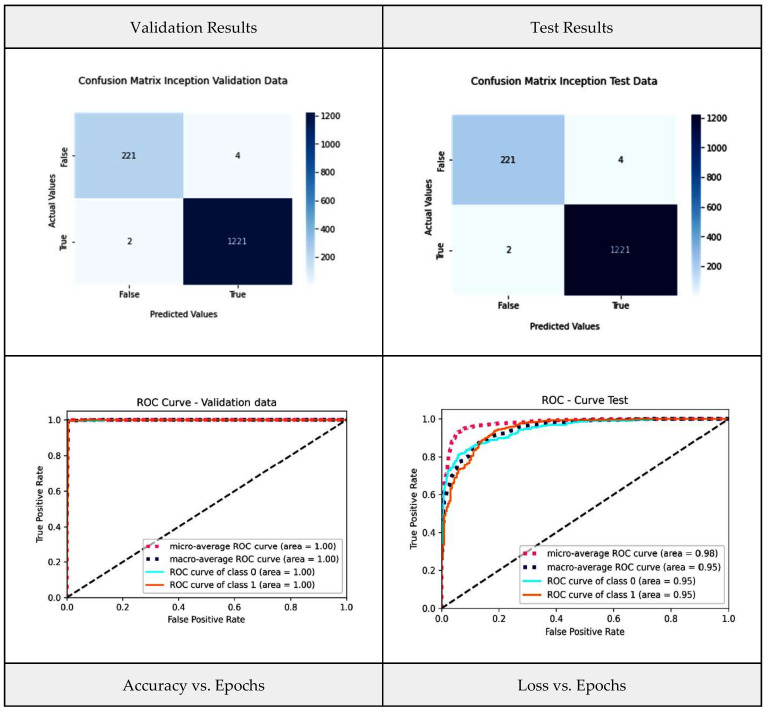

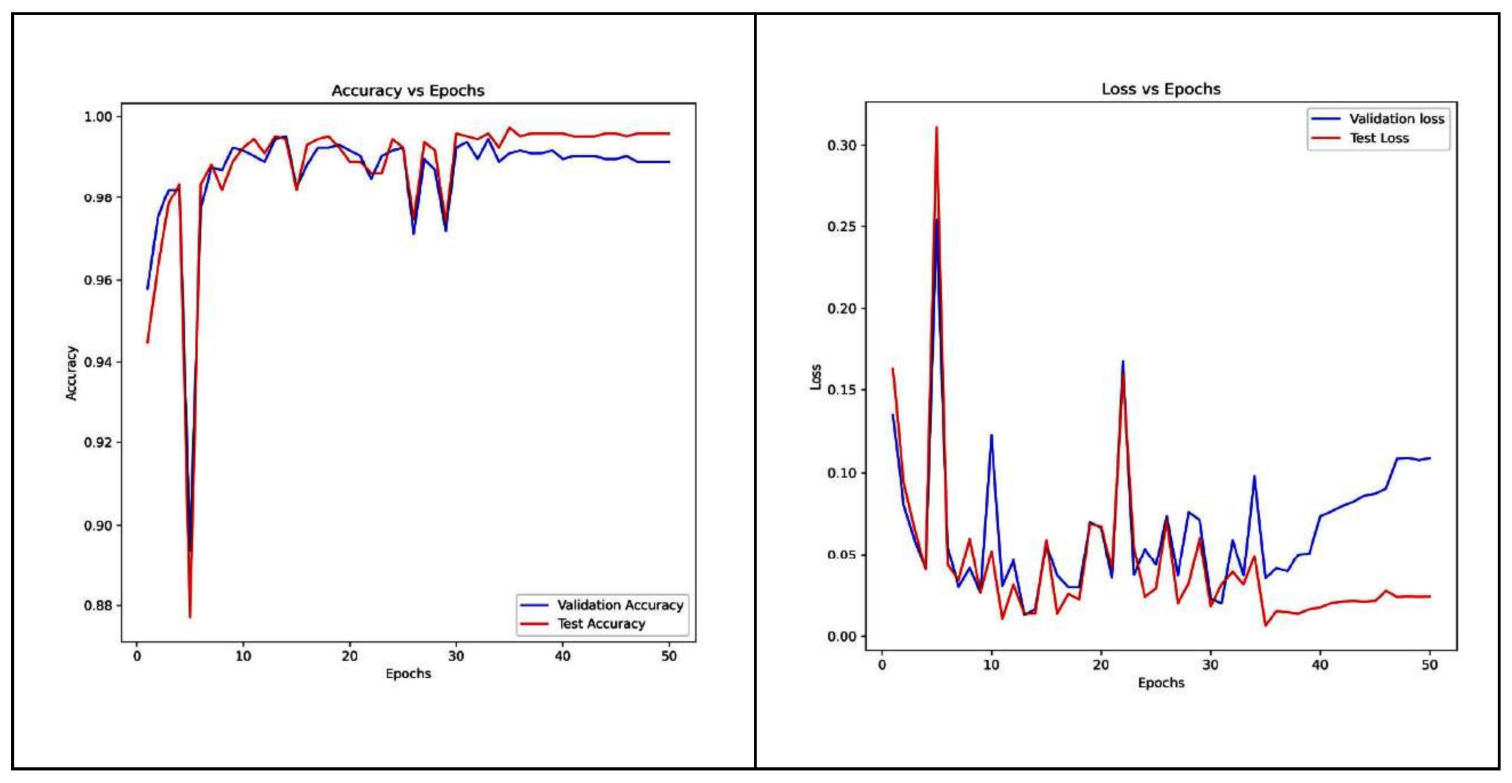
Inception net model performance metrics.

The inception network is one of the novel models in the field of transfer learning. The architectural design of the model consists of repeating components referred to as inception modules [44], and it is represented in Figure 8. Figure 9 shows the inception model performance metrics, such as the confusion matrix and the ROC for the validation and test data, as well as graphs for accuracy vs. epochs and loss vs. epochs.

The model was trained using Adam [lr = 0.001], dropout [probability = 0.2], and a dense layer of [1000, 75, 2]. We have used callbacks that have a patience of 5 and monitored value_loss while training.

After training over these parameters, the best model metrics are:

- Epochs: 8/30.
- Callbacks: Early stopping on val_accuracy with patience 5, restore best weights true.
- Optimizer: Adam with learning rate of 0.001.
- Loss: BinaryCrossEntropy without logits.
- Image_size: (224 × 224 × 3), type: tensor.
- Dense Layer Weight—Initializer: glorot_uniform.
- Activation: Relu.
- Use Bias: True.
- Dense Layer Bias—Initializer: zeros.
- Batch Normalization: a momentum of 0.99 with an epsilon of 0.001 along with gamma-initializer of ones, a beta-initializer of zeros and a moving mean initializer of zeros.

### 4.4. Capsule Net (CapsNet) Model

**Figure 10:**
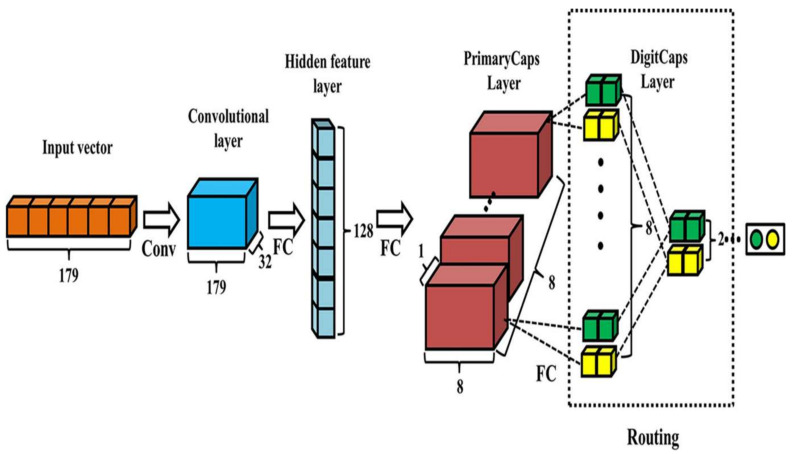
CapsNet model architecture.

A CapsNet consists of capsules as compared to neurons. A capsule is a tiny group of neurons that learns to recognize a certain object within a particular visual region, and it produces a vector. It is structured in several layers. The capsules in the lowest layer are referred to as primary capsules. Each of these capsules receives a small visual region as input and attempts to detect the presence and orientation of a specific pattern. Higher-layer capsules, known as routing capsules, detect larger and more complicated objects [45]. The layer details are represented in Figure 10.

The model weights were initialized by glorot_uniform and they were optimized during the training phase. The model was trained using Adam [lr = 0.001]. We used callbacks that have a patience of 5 and tracked value_loss while training.

After training over these parameters, the best model metrics are:

Primary capsules are the first layer of capsules in the network and aim to capture distinctive features in the input data. Capsules is a module list containing eight primary capsules. Each primary capsule consists of:

- Input: 256 channels (output from the previous convolutional layer).
- Output: 32 capsules.
- Convolutional Operation: 2D convolution with a 9 × 9 kernel and a stride of 2. Each primary capsule performs this operation.
- Digit Capsules: (digit_capsules).
- Decoder
- The decoder component is responsible for reconstructing the input data from the output of the capsule network. It aims to create a reconstruction of the original input.

The decoder consists of a series of linear layers and non-linear activation functions:

- Linear Layer 1:
- Input: 160-dimensional capsule output.
- Output: 512 units.
- ReLU Activation: Applies the Rectified Linear Unit activation function.
- Linear Layer 2:
- Input: 512 units.
- Output: 1024 units.
- ReLU Activation: Another ReLU activation.
- Linear Layer 3:
- Input: 1024 units.
- Output: 784 units.
- Sigmoid Activation: Applies the sigmoid activation function to create the final reconstruction.

In summary, this capsule network architecture consists of a convolutional layer to extract features, primary capsules to capture primary features, digit capsules for higher-level recognition (architecture not detailed in the code), and a decoder to reconstruct the input from the capsule network’s output.

**Figure 11:**
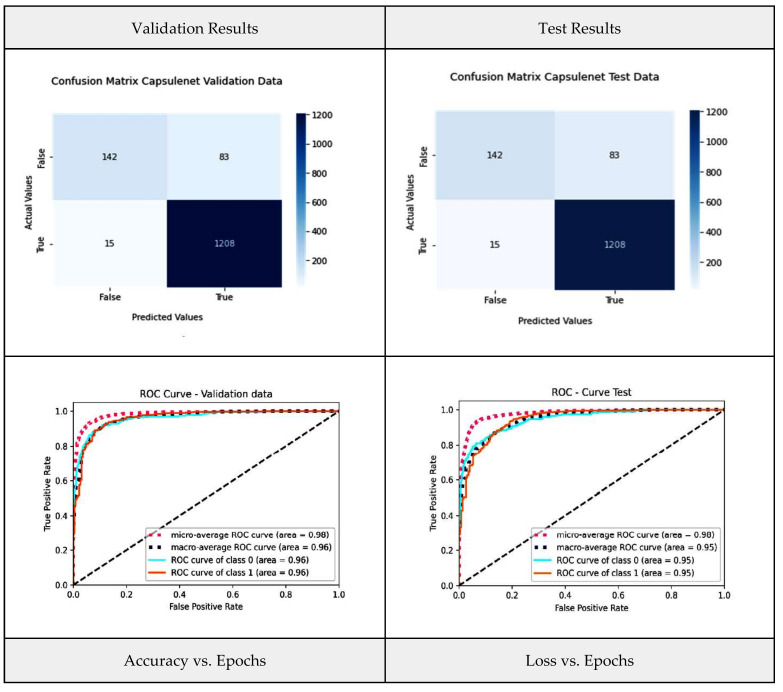

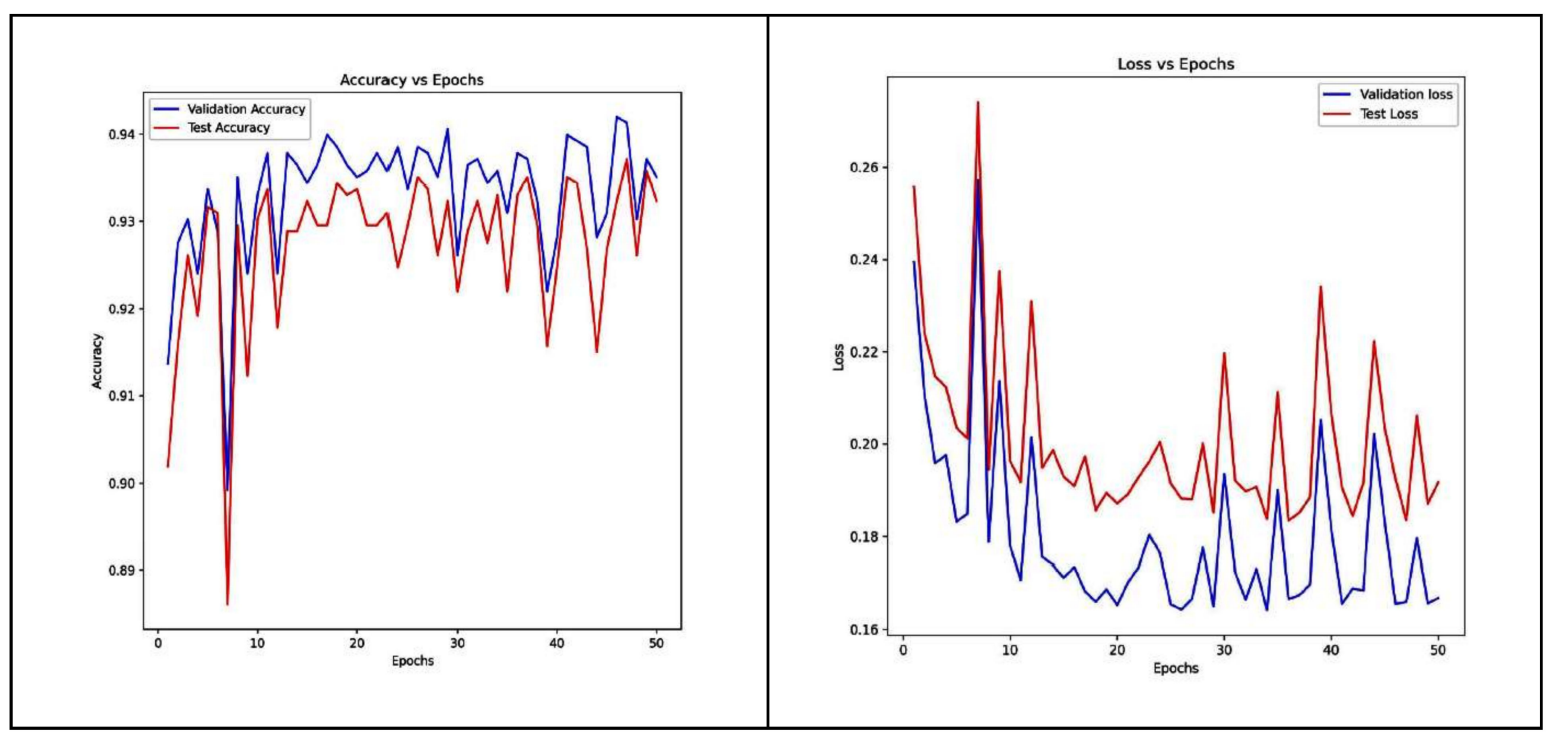
CapsNet model performance metrics.

In our present study, CapsNet represents the base model that accepts the CT-scan images and original labels. In a later phase, its output is used as an input to the aggregator model. Figure 11 shows the CapsNet model performance metrics, such as the confusion metrics and the ROC for the validation and test data, as well as graphs for accuracy vs. epochs and loss vs. epochs. From Figure 11, it is revealed that CapsNet produced few classifications as compared to the transfer learning models. Ninety-eight images are misclassified, as eighty-three images are categorized as false-positive. The AUC-ROC curve manifests an AUC value of 0.96 for validation data.

**Figure 12:**
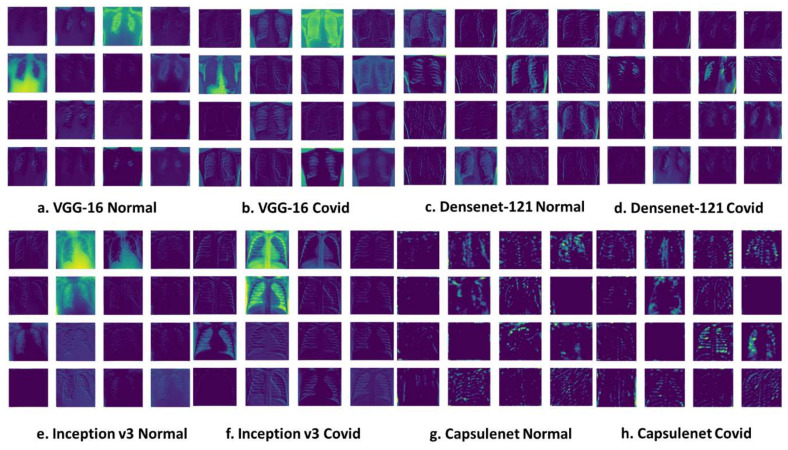
Feature maps generated by transfer learning models.

Figure 12 shows a feature map for COVID and normal images as generated by VGG-16, Densenet-121, Inception V3 and CapsuleNet, respectively.

## 5. Results and Interpretation

The model was trained for 50 epochs with 32 batch sizes or 331 images for each epoch; the loss was then back-propagated with the assistance of the Adam optimizer. To safeguard the model from overfitting, we implemented the call-back with five epochs of tolerance on the validation and test accuracy maximum value. If the given measure did not improve within five epochs, the training of the model was terminated, and the weights were reset to a minimum value of the difference between the test and validation accuracy. This resetting refers to a stopping criterion used during the training of the model, particularly during techniques like early stopping, where training is halted if a certain condition is met. In early stopping, the performance of the model is monitored on a validation dataset during training, and a “patience” threshold can be set, which is the number of epochs or iterations during which the validation accuracy can decrease or not improve by a definite amount. If the difference between the validation accuracy and the best validation accuracy obtained during training exceeds a certain threshold, training is stopped, and the model weights are reset to the point where the validation accuracy was the best.

**Figure 13:**
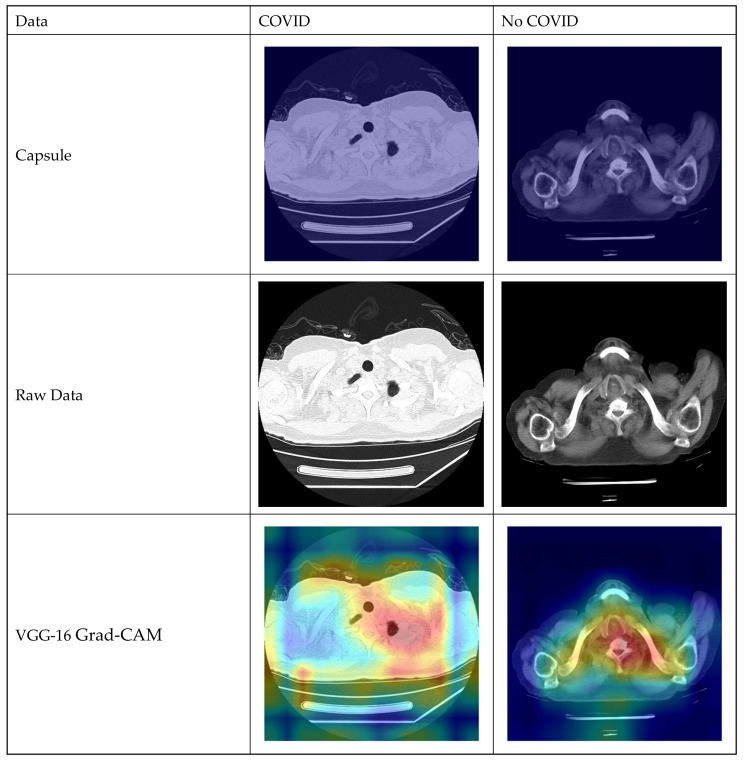

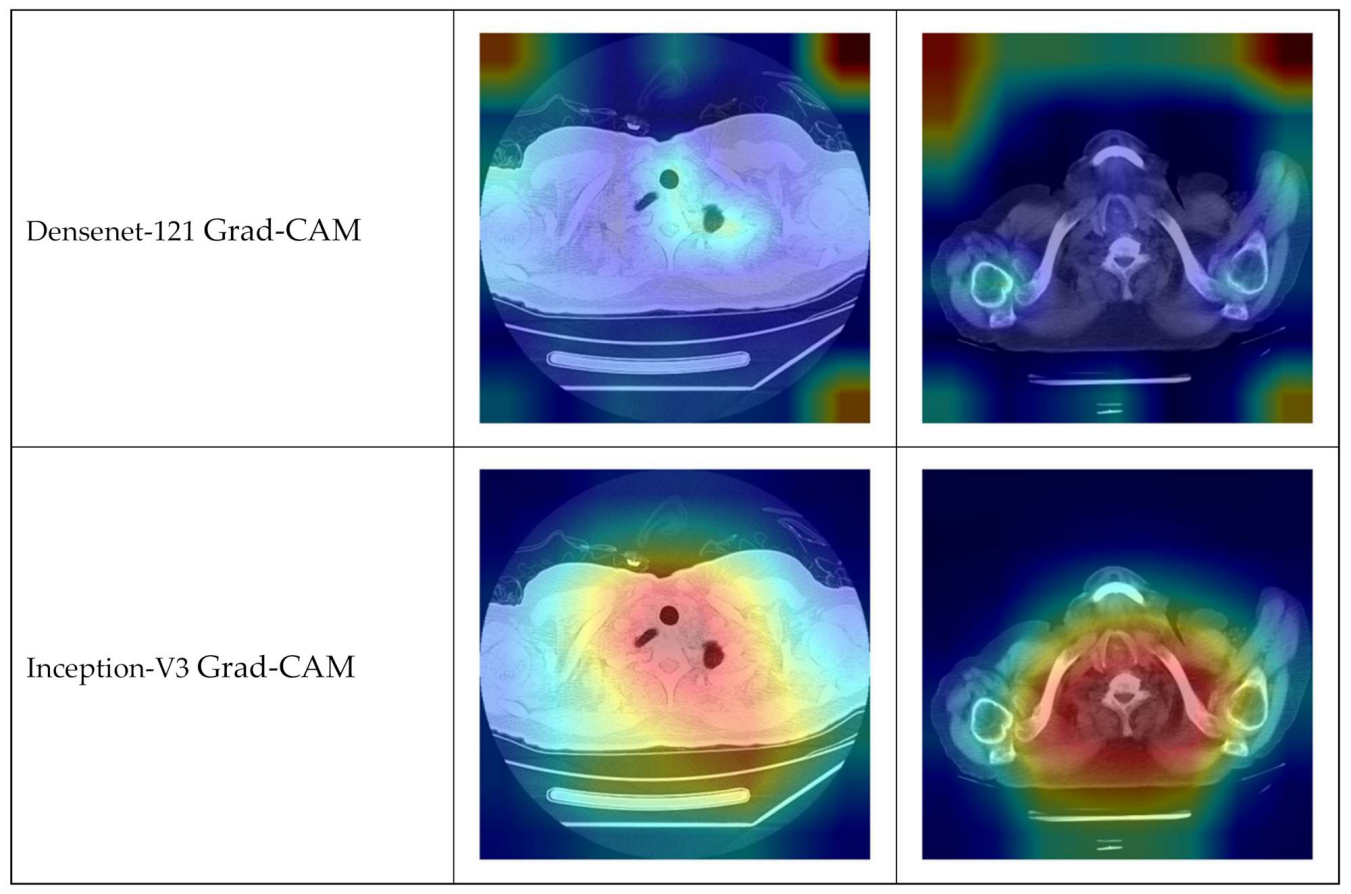
Grad-CAM of the models.

Grad-CAM (Gradient-weighted Class Activation Mapping) is a valuable technique in the context of COVID-19 classification using transfer learning and capsule networks (CapsuleNet). In this scenario, the primary task is to determine whether CT-scan images of a patient’s lungs exhibit signs of COVID-19 or not. Transfer learning plays a pivotal role by leveraging pre-trained models, such as inception, which have learned rich features from extensive datasets like ImageNet. These models serve as a foundation for the COVID-19 classification task, allowing for the adaptation of their knowledge to a smaller and specific COVID-19 dataset. The Grad-CAM results are represented in Figure 13.

Capsule networks, known for capturing hierarchical feature relationships in images, may be used in place of conventional convolutional neural networks (CNNs) for feature extraction and classification. In the context of Grad-CAM, it serves as an invaluable tool to visualize which areas of the X-ray or CT scan significantly influence the COVID-19 classification decision. This visualization aids in the interpretation of model predictions, allowing healthcare professionals to confirm whether the model’s attention aligns with the expected regions associated with COVID-19 symptoms in the medical images. The synergy of Grad-CAM, transfer learning, and CapsuleNet contributes to the model’s transparency and trustworthiness in critical healthcare applications, where interpretability is of paramount importance for medical practitioners.

We employed ensemble learning in accordance with deep learning, where the basic learners are pre-trained models such as VGG-16, Inception-v3, Densenet-121, and CapsNet. Table 3 shows the complete model parameters for both the base learners and the aggregators, such as stacking and weighted average.

Ensemble modeling is the creation of numerous diverse models to forecast an outcome, either by utilizing a variety of modeling algorithms or by utilizing various training datasets. Subsequently, the ensemble model combines the outcomes of each base model’s forecast to provide a single, conclusive prediction for the unseen data. Ensemble learning is used to improve the prediction’s accuracy and reliability [46].

### 5.1. Stacked Generalization (Stacking)

**Figure 14:**
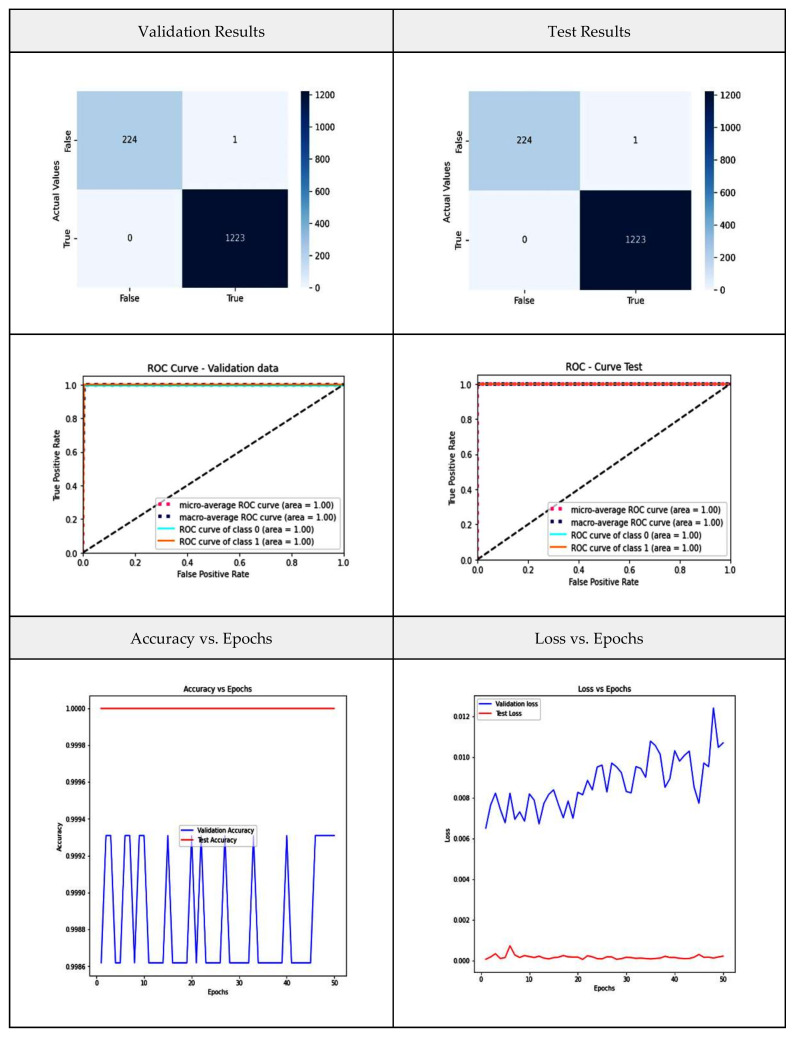
Stacking model performance metrics.

Model stacking is a technique for enhancing model predictions by aggregating the results of various models and passing them through a meta-learner, which becomes another machine learning model for prediction [47]. Figure 14 shows ensemble stacking model performance metrics, such as the confusion metrics and the ROC for the validation and test data, as well as graphs for accuracy vs. epochs and loss vs. epochs.

### 5.2. Weighted-Average Ensemble (WAE)

Weighted averaging is an ensemble learning technique in which each participant contributes equally to the prediction. In contrast, when using weighted model averaging, outcomes are taken into account according to the relative relevance of each of the base learners. Typically, the base learners’ top performers will receive the higher weight. In our experiment, the weights are distributed as follows: Densenet = 0.8, VGG = 0.6, Inception = 0.4, and Capsule = 0.2.

**Figure 15:**
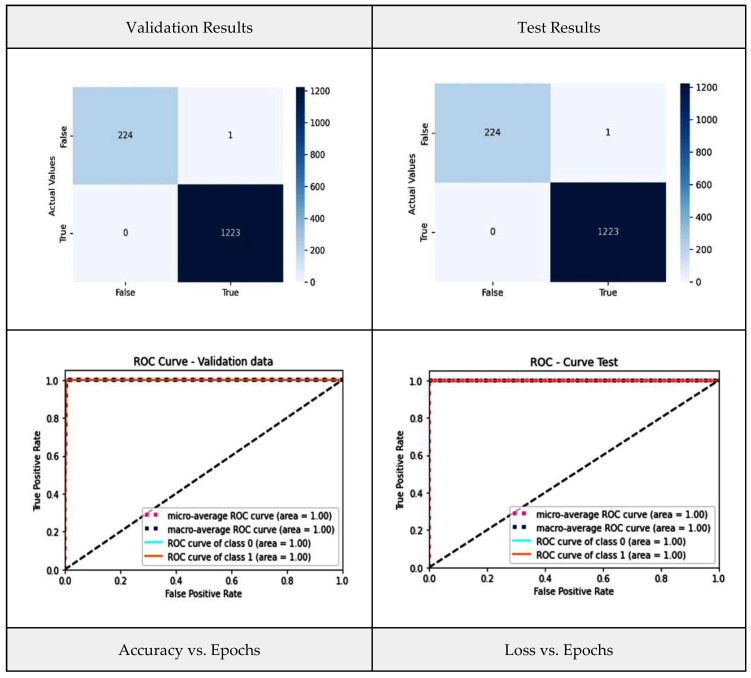

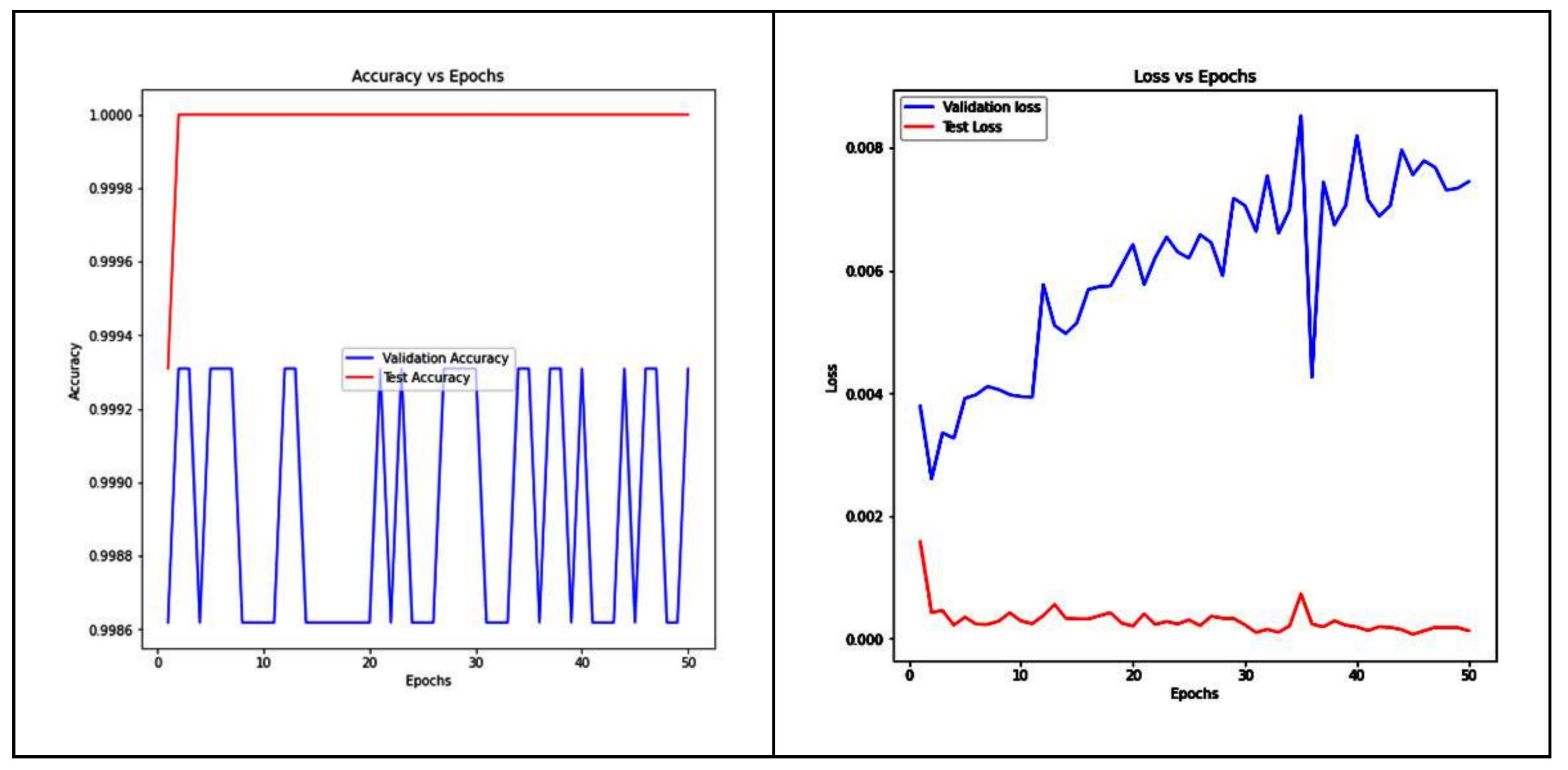
Weighted-average model performance metrics.

Figure 15 shows the ensemble weighted-average model performance metrics, such as the confusion matrix and the ROC for the validation and test data, as well as graphs for accuracy vs. epochs and loss vs. epochs.

The confusion matrix reveals that only one instance is misclassified, showing the highest ever classification accuracy of 99.9%. The accuracy of the model steeply increases with each epoch, and after five epochs, it converges to the maximum level and becomes stable.

### 5.3. Performance Analysis of Proposed Ensemble Models and Base-Learners

Table 4 shows the test results of all the base learners (VGG-16, Inception, DenseNet, CapsNet) and the ensemble methods (stacking and weighted average). Among the base learners, inception produces the highest accuracy of 98.78% and an AUC value of 0.997 for the validation and test data. However, both the ensemble methods, e.g., stacking and weighted average, generate an accuracy of 99.9%.

Since the number of trainable images is high, we used a stacking technique for the ensemble. However, in order to minimize the bias, we also formed one more ensemble model by averaging the weight given by the individual models.

**Figure 16:**
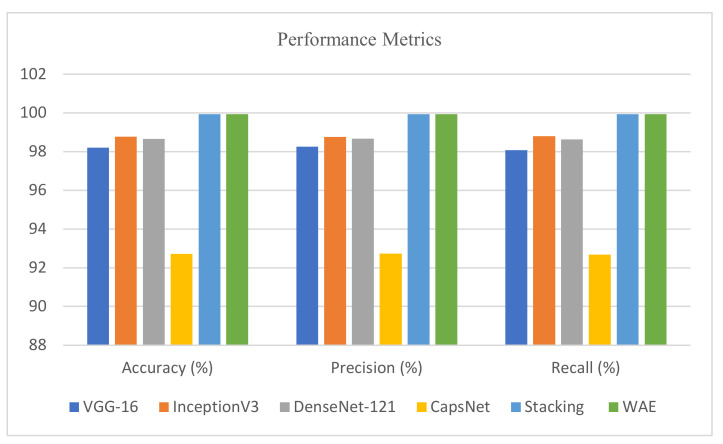
Comparison of accuracy, precision and recall of all six models.

We consolidated the average accuracy, average precision and average recall attained by base learners and our proposed ensemble models, and the results are exhibited in Figure 16. It is clear from the figure that both the stacking and WAE models achieved almost equal accuracy, precision and recall values of 99.9%. Hence, both the models are equally competent in detecting the COVID variants. Thus, this signifies that training the images using either of the ensemble methods boosts the classification performance; therefore, the model is reliable and can be employed for tracing the virus strain in lungs.

The proposed approach can also be used for other conditions such as detecting pneumonia and collapsed lungs from chest X-rays, diagnosing certain cancers from CT scans, and tracing brain tumors using MRI.

## 6. Conclusions

This paper researches the usage of deep ensemble learning models for diagnosing COVID-19 Delta and Omicron variants. At first, we employed three standard transfer learning models, and the fourth one is a relatively new model that is not widely explored yet: CapsNet. Next, we proposed two ensemble learning models: (i) stacking (ii) weighted-average ensemble. Each of the ensemble models was set up by combining VGG16, DenseNet201, Inceptionv3, and CapsNet. For training and validating the models, we employed Omicron and Delta variant CT-scan images collected from the Kaggle platform. Before the ensemble, we fine-tuned the pertained models along with CapsNet. The experimental results obtained are of the highest levels, as we achieved an accuracy of 99.9%, a precision and recall of 99.9% each, and an AUC of 0.999. The results signify that there is absolutely no instance of the misclassification of images; hence, either of the ensemble models is recommended for diagnosing COVID-19 Delta and Omicron variant infection using lung CT-scan images. However, in order to obtain further explanation of the prediction, an explainable intelligent system with the argumentation feature can be explored [48].

It is well known that the Delta variant was mostly dreadful and was a killer variant. Even though we were successful in detecting the variant from the chest images, the assessment of its severity from the images has to be realized. In future, our proposed approach can be employed in patient-centric systems involving physicians for real-time analysis and diagnosis similar to the SIMPATICO 3D Mobile system [49]. The presence of the variant in a patient along with its severity and farsighted consequences will be valuable for the healthcare fraternity in terms of dealing with that particular COVID variant.

## Figures and Tables

**Table 1:** Review of methods and quantitative results for the classification of COVID-19 X-ray and CT-scan images using deep learning and ensemble methods.

Studies	Objective	Data Description	Methodology	Key Findings
Chouhan et al., 2020 [11]	To increase the accuracy of an ensemble model	Chest X-ray images.	Uses five different transfer learning models and the results are aggregated.	This research has resulted in an accuracy of 96.4% and a recall of 99.62%, respectively.
Jaiswal et al., 2021 [12]	To enhance classification accuracy of the model	SARS-CoV-2 CT scan dataset.	Uses a deep transfer learning model DenseNet20.	This research has resulted in an accuracy of 97%.
Arora et al., 2021 [13]	The objective of the paper is to increase the accuracy of the classification	SARS-CoV-2 CT scan and COVID-CT scan.	This study fine-tunes a variety of pre-trained models, including VGG (16, and19), MobileNet, and ResNet (50, and 50V2), InceptionResNetV2, Xception, InceptionV3.	Model performance has resulted in a precision of 94.12% and 100%.
Singh et al., 2021 [14]	The objective is to predict the early-stage detention of COVID-19 using chest CT.	To maintain the robustness of the model, the dataset is gathered from three independent sources.	DCNN, ELM, online sequential ELM, and bagging ensemble with SVM-these four classifiers are compared for the final classification.	Bagging ensemble with SVM is the leading performer, achieved an F1 score of 0.953, accuracy of 95.7%, precision of 0.958, and AUC of 0.958.
Gifani et al., 2021 [15]	To provide accurate and fast detection of COVID-19 using CT-scan images.	Publicly available datasets.	The overall classification decision is made based on the assembly ensemble of all the models using the majority voting method, using 15 pre-trained CNN architectures.	Accuracy: 0.85, precision: 0.857, recall: 0.852.
Kundu et al., 2022 [16]	The objective is to increase the accuracy of an ensemble model consisting of 3 different transfer learning techniques.	Publicly available datasets on GitHub.	Ensembling of three transfer learning models, i.e., DenseNet201, Inception v3, and, ResNet34 has been utilized.	Research impact resulting in a 97.77% accuracy rate.
Shaik et al., 2022 [17]	The objective is to improve prediction accuracy and reliability.	SARS-CoV-2 and COVID-CT chest CT-scan image datasets.	The ensemble approach is carried out using 8 different pre-trained models.	The highest accuracy on the SARS-CoV-2 dataset is 98.9% COVID-CT dataset is 93.3%.
Khan et al., 2022 [18]	To design a model for predicting any sort of variant and its potential health risks.	Publicly available X-ray images on GitHub.	The VGG16 architecture is used in handling the classification tasks and the SVM is used in handling statistical analysis to determine the severity of the patient’s health condition.	The obtained accuracy is 97.37%.
Vocaturo et al., 2021 [19]	COVID-19 classification detection from chest X-rays by CNN	COVID dataset containing 13.800 chest X-ray images.	ResNet50.	Accuracy of 98.66%
Rani et al., 2022 [20]	COVID-19 detection using chest X-rays	COVID Pneumonia CXR, which includes bone-suppressed and lung-segmented chest X-rays.	GAN and CNN.	AUC of 96.58% on the validation dataset and 96.48% on the testing dataset
Zumpano et al., 2021 [21]	Viral and bacterial pneumonia detection	Chest X-ray dataset from Kaggle.	Multiple instance learning paradigm.	Accuracy about 90% and sensitivity about 94%.

**Table 2:** Experimental dataset distribution.

Dataset	COVID Images	Non-COVID Images	Total
Kaggle Dataset	12,231	2251	14,482

**Table 3:** Parameters of transfer learning and ensemble learning models.

Name of the Model	Number of Layers	Fully CLayer	Classifier Activation	Optimizer
Common attributes of all the models: Data Split: Train, Validation, Test Loss Function: Binary Cross Entropy Activation Function: ReLU Epochs: 50				
Base Learners				
VGG-16	16 (all trainable)	256, 64, 32, 1	Sigmoid	Adam (lr = 0.001)
Densenet-121	121 (all trainable)	256, 64, 32, 1	Sigmoid	Adam (lr = 0.001)
Inception-v3	22 (all trainable)	256, 64, 32, 1	Sigmoid	Adam (lr = 0.001)
CapsNet	14	256, 64, 32, 1	Sigmoid	Rmsprop (lr = 0.001)
Ensemble Models				
Stacking	7	10, 4, 1	Sigmoid	Adam
Weightage Average Aggregator Densenet = 0.8 VGG = 0.6 Inception = 0.4 Capsule = 0.2	7	10, 4, 1	Sigmoid	Adam

**Table 4:** Comparison of ensemble models’ performances with base learners’ performances.

Model Name	Accuracy (%)	Precision (%)	Recall (%)	AUC
Base Learners				
VGG-16	98.21	98.26	98.08	0.9924
InceptionV3	98.78	98.77	98.8	0.997
DenseNet-121	98.66	98.67	98.64	0.996
CapsNet	92.72	92.74	92.69	0.9747
Ensemble Methods				
Stacking	99.93	99.93	99.93	0.9993
WAE	99.93	99.93	99.93	0.9993

## Data Availability

The dataset analyzed during the current investigation is available in the following repository: https://www.kaggle.com/datasets/mohammadamireshraghi/covid19-omicron-and-delta-variant-ct-scan-dataset (accessed on 30 November 2022).
